# The burden of laboratory-confirmed pertussis in low- and middle-income countries since the inception of the Expanded Programme on Immunisation (EPI) in 1974: a systematic review and meta-analysis

**DOI:** 10.1186/s12916-020-01699-3

**Published:** 2020-08-28

**Authors:** Rudzani Muloiwa, Benjamin M. Kagina, Mark E. Engel, Gregory D. Hussey

**Affiliations:** 1 Department of Paediatrics & Child Health, Groote Schuur Hospital, University of Cape Town, Main Road, Observatory, 7925 Cape Town, Republic of South Africa; 2 Vaccines for Africa Initiative, School of Public Health and Family Medicine, University of Cape Town, Anzio Road, Observatory, 7925, Cape Town, Republic of South Africa; 3 Department of Medicine, Groote Schuur Hospital, University of Cape Town, Main Road, Observatory, 7925, Cape Town, Republic of South Africa; 4 Division of Medical Microbiology & Institute of Infectious Disease and Molecular Medicine, University of Cape Town, Anzio Road, Observatory, 7925, Cape Town, Republic of South Africa

**Keywords:** Pertussis, Burden, Prevalence, Incidence, Mortality, Case fatality, HIV, Low- and middle-income countries (LMIC)

## Abstract

**Background:**

An effective vaccine against *Bordetella pertussis* was introduced into the Expanded Programme on Immunisation (EPI) by WHO in 1974, leading to a substantial global reduction in pertussis morbidity and mortality. In low- and middle-income countries (LMICs), however, the epidemiology of pertussis remains largely unknown. This impacts negatively on pertussis control strategies in these countries. This study aimed to systematically and comprehensively review published literature on the burden of laboratory-confirmed pertussis in LMICs over the 45 years of EPI.

**Methods:**

Electronic databases were searched for relevant literature (1974 to December 2018) using common and MeSH terms for pertussis. Studies using PCR, culture or paired serology to confirm *Bordetella pertussis* and *parapertussis* in symptomatic individuals were included if they had clearly defined numerators and denominators to determine prevalence and mortality rates.

**Results:**

Eighty-two studies (49,167 participants) made the inclusion criteria. All six WHO regions were represented with most of the studies published after 2010 and involving mainly upper middle-income countries (*n* = 63; 77%). PCR was the main diagnostic test after the year 2000.

The overall median point prevalence of PCR-confirmed *Bordetella pertussis* was 11% (interquartile range (IQR), 5–27%), while culture-confirmed was 3% (IQR 1–9%) and paired serology a median of 17% (IQR 3–23%) over the period. On average, culture underestimated prevalence by 85% (RR = 0.15, 95% CI, 0.10–0.22) compared to PCR in the same studies.

Risk of pertussis increased with HIV exposure [RR, 1.4 (95% CI, 1.0–2.0)] and infection [RR, 2.4 (95% CI, 1.1–5.1)]. HIV infection and exposure were also related to higher pertussis incidences, higher rates of hospitalisation and pertussis-related deaths.

Pertussis mortality and case fatality rates were 0.8% (95% CI, 0.4–1.4%) and 6.5% (95% CI, 4.0–9.5%), respectively. Most deaths occurred in infants less than 6 months of age.

**Conclusions:**

Despite the widespread use of pertussis vaccines, the prevalence of pertussis remains high in LMIC over the last three decades. There is a need to increase access to PCR-based diagnostic confirmation in order to improve surveillance. Disease control measures in LMICs must take into account the persistent significant infant mortality and increased disease burden associated with HIV infection and exposure.

## Background

Pertussis is a highly infectious respiratory illness caused by *Bordetella pertussis* or *Bordetella parapertussis*. Using a model developed in 2003, the World Health Organization (WHO) estimated that in 2008 there were 20 to 40 million annual cases of pertussis. They further estimated that 90% of the cases and their 300,000 associated deaths occurred in low- and middle-income countries (LMICs) [1, 2]. An updated model by WHO estimated that 24.1 million cases of pertussis occurred in 2014 with 160,700 associated deaths in children under 5 years of age; a majority of these (58%) were estimated to have occurred in the African region and largely involved infants (53%) [3].

While there are good surveillance data to support the re-emergence of pertussis in high-income countries (HICs), the disease trends are unknown in LMICs due to the paucity of epidemiological data in these settings [4, 5]. A non-systematic review of available data for the African continent was published recently by the Global Pertussis Initiative (GPI) [6].

The high HIV prevalence estimates in LMICs coupled with suboptimal vaccine uptake are modifiable risk factors that can fuel high pertussis burdens [7, 8]. The pertussis resurgence reported lately in HICs has resulted in the review of disease control strategies in these countries [4, 9]. A review of existing pertussis control programmes in LMICs is yet to be undertaken.

The availability of an effective vaccine against *Bordetella pertussis* since the 1940s has led to a substantial global reduction in the morbidity and mortality caused by pertussis [10]. In 1974, WHO included the whole cell vaccine (wP) in the Expanded Programme on Immunisation (EPI) adopted in several countries. Although wP is still widely used in many LMICs, many HICs have replaced wP with various formulations of the acellular vaccine (aP) [11]. Epidemiological data from HICs show that despite high vaccine coverage with aP, the pertussis burden has increased in non-immunised and partially immunised infants, as well as in previously immunised adolescents and adults [4, 9, 12–14]. The reported pertussis resurgence has been linked to several factors such as reduced efficacy of aP vaccines and genetic evolution of the pertussis bacteria as well as improved diagnosis and reporting of the disease [5].

A sound understanding of trends in the burden of pertussis is required to assess the impact of current pertussis control strategies as well as to decide on future policy. We conducted a comprehensive systematic review to address the knowledge gap in the longitudinal epidemiology of pertussis in LMICs for the 45 years starting in 1974 to 2018, inclusive. Primarily, our systematic review aimed to review available published literature on the prevalence and/or incidence of laboratory-confirmed pertussis in LMICs since the inception of the EPI and to determine the trend in the burden of pertussis in LMICs from 1974. For secondary objectives, we sought to determine the mortality and case fatality rates ascribed to pertussis in LMICs, as well as to investigate the impact of vaccine choice, and HIV infection and in utero exposure on the burden of pertussis in LMICs over the review period.

## Methods

### Search strategy and criteria for selecting studies

The protocol for the systematic review was registered with PROSPERO International Prospective Register of systematic reviews (http://www.crd.york.ac.uk/PROSPERO), with registration CRD42015015159. The methods employed in conducting this review have been previously published [15]. The following electronic databases were searched for qualifying literature: MEDLINE, Scopus, Africa-Wide, PDQ-Evidence, WHOLIS, CINAHL, CENTRAL and Web of Science. Search terms used included “pertussis,” “*Bordetella pertussis*”, “*Bordetella parapertussis*” and “whooping cough” combined with “burden”, “epidemiology”, “incidence”, “prevalence” and “case”. These were used together with the specific names of all LMICs as classified by the World Bank [16, 17]. The search strategy as used in MEDLINE via PubMed is shown in Additional file 1. The reported search was carried out in April 2019. The World Bank groupings reflect the country status at this period.

The search was limited to studies published from 1974, the year that the EPI was introduced, until December 2018. Titles and abstracts of the search outputs and references were screened, and the full texts of potentially relevant articles were independently assessed by two reviewers (RM and BK) using a standardised score sheet. Disagreements on final inclusions were resolved by consensus following discussions involving a third reviewer (GH). Authors and publishers were contacted for full texts not available online or via our collaborative networks.

Studies were included if the study populations were from LMICs. While the diagnosis of pertussis is largely made on the basis of clinical parameters, it is well-known that clinical presentation may be modified by age, previous immunisation or infection, antibiotic exposure and concurrent infection with other pathogens [10]. This makes the presentation of pertussis frequently atypical, thus requiring laboratory confirmation of cases by serology, culture or polymerase chain reaction (PCR). Therefore, laboratory confirmation by either PCR, culture or paired serological assays was also an inclusion criterion.

Studies that failed to provide a numerator (number of participants testing positive) or denominator (number of participants tested for pertussis), as well as those that failed to specify the laboratory diagnostic method utilised, were excluded. Studies on sero-epidemiological and laboratory diagnostic methods in the absence of clinical disease were also excluded.

The systematic review included published randomised controlled trials, cross-sectional, cohort and surveillance studies. Case series and review articles were excluded as they failed to provide the required denominator.

### Data extraction

The denominator and numerator were extracted from each study to determine the prevalence for each diagnostic method. We defined prevalence as proportions with confirmed laboratory diagnosis from all participants suspected and tested for pertussis. *Bordetella pertussis* prevalence data were stratified by WHO region, diagnostic method (culture, paired serology or PCR), clinical setting (hospital or population based) and age category of the study participants. Prevalence was further stratified by HIV status, that is HIV infected (HIV+), HIV-exposed uninfected (HEU) and HIV-unexposed uninfected (HUU). Incidence data were extracted as reported by the authors.

The epidemiology of *Bordetella parapertussis* was separately assessed. Data on the type of pertussis vaccine (wP or aP) used, clinical diagnostic criteria (e.g. WHO, Centers for Disease Control and Prevention (CDC), etc.) and the study design were captured.

Foreign language articles were reviewed, and data extracted with the assistance of online translation programmes and native speakers [18, 19].

### Data analysis and reporting

Percentage point estimates together with their 95% confidence intervals (CIs) were calculated to represent the prevalence of laboratory-confirmed pertussis for all outcomes. The Mantel-Haenszel method was used to pool together prevalence data from individual studies using random-effects meta-analysis. Heterogeneity was evaluated both visually by assessing forest plots and formally using the *χ*^2^-based *Q* and *I*^2^ statistics [20]. Where a meta-analysis was not feasible, either because data were too heterogeneous or insufficient to allow for meaningful pooling, narrative reporting was used.

Narratively reported frequencies were summarised using medians and interquartile ranges (IQR) of prevalence point estimates and graphically represented using forest-like plots that omitted pooled data. Instead, dotted lines were used to indicate where group averages would lie without emphasising their meaning. The Kruskal-Wallis test was used to compare point prevalence between groups.

Mortality was defined as the proportion of deaths attributable to pertussis in the study sample while case fatality was defined as mortality attributable to the disease among confirmed cases of pertussis.

The incidence of pertussis could not be independently estimated as the requisite data was not available. Incidence was narratively reported per 100,000 as reported by the authors themselves.

All statistical analyses were done on STATA software version 14 (STATA Corporation, College Station, TX). The command *metaprop_one* was used to generate pooled prevalence forest plots after Freeman-Tukey transformation and *metan*, for comparative effect forest plots showing relative risks (RR) and their 95% CI, respectively.

The study utilised the guidelines for reporting systematic reviews as set down by the revised 2009 PRISMA Statement [21].

### Quality of included studies

An adaptation of the tool developed by Wasserman et al. was used to assess the risk of bias as well as the quality of the included studies [22]. The quality assessment criteria examined specific variables to make judgement on the studies, taking into account methodological aspects discussed by Hoy et al. pertaining to internal and external validity of prevalence studies [23].

## Results

### Characteristics of the included studies

**Fig. 1:**
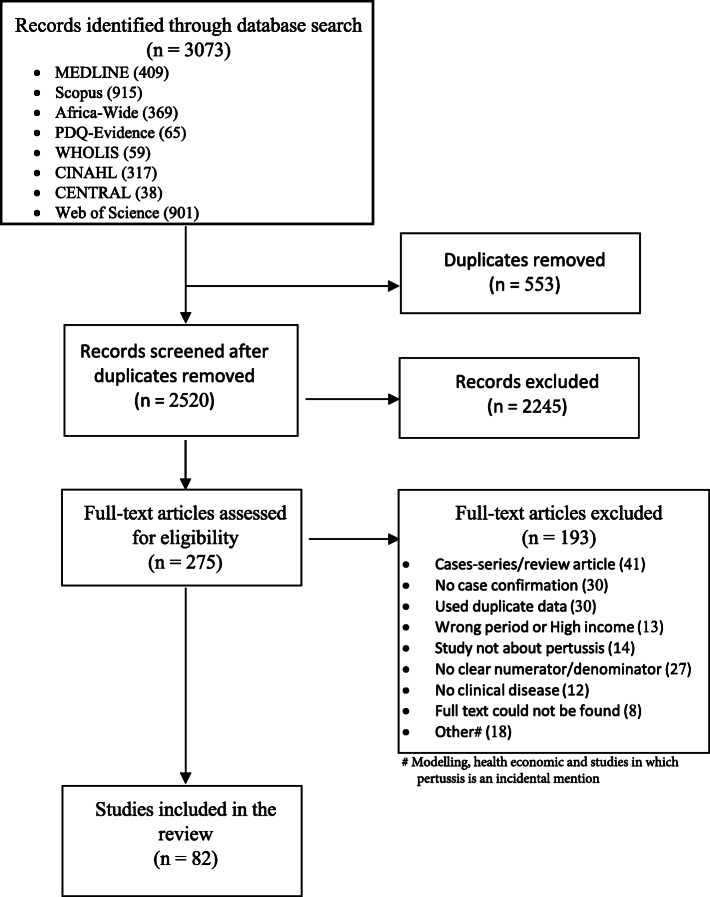
Studies included in the systematic review

The search strategy returned 3186 studies which reduced to 2633 after excluding duplicates. Following screening of abstracts and titles, 275 articles were deemed potentially relevant and subjected to full-text evaluation. Eighty-two studies (*n* = 49,167) met the final criteria for inclusion into the systematic review (Fig. 1). Studies were excluded if they did not report clinical cases such as in laboratory studies, animal studies, economic evaluation and modelling studies. Other reasons for exclusions are shown in Fig. 1. The included studies involved symptomatic individuals meeting WHO and CDC (*n* = 52 and *n* = 8 respectively) clinical criteria. The remaining studies (*n* = 22) used clinical definitions derived from modifications of the criteria set by WHO or CDC. Two studies were multinational (two and seven countries in each) so that in the end the final 82 studies included, represented 88 unique populations [24, 25].

Table 1 shows the characteristics of the included studies by WHO region. A large proportion of the studies (*n* = 63; 77%) was published between 2010 and 2018. Sixty-eight studies (83%) involved hospital-based participants. Forty-seven studies (57%) used one laboratory diagnostic confirmatory test while 32 (39%) and three (4%) studies used two and three methods for pertussis diagnosis, respectively.

**Table 1:** Characteristics of studies included in the systematic review

Region and study	Design	Setting	Diagnosis	Country	Vaccine	Period	Sample (cases)
**Africa**							
Voorhoeve^#^ (1978) [26]	Surveillance	Population	C, S	Kenya	wP	1974–19077	1078 (138)
Ramkissoon (1991) [27]	Clinical trial	Population	S	South Africa	wP	1988	112 (3)
Strebel (1991) [28]	Surveillance	Hospital	C, S	South Africa	wP	1989	34 (3)
Simondon (1997) [29]	Clinical trial	Population	C, P, S	Senegal	wP and aP	1990–1995	3619 (193)
Anukam^#^ (2004) [30]	Cross-sectional	Hospital	C	Nigeria	wP	1997–2000	296 (22)
Lassmann (2008) [31]	Cross-sectional	Hospital	P	Gabon	wP	2003–2004	99 (6)
Jusot^#^ (2014) [32]	Cross-sectional	Hospital	C, P	Niger	wP	2010–2011	305 (34)
Kayina (2016) [33]	Cross-sectional	Hospital	P	Uganda	wP	2013	449 (67)
Barger-Kamate (2016) [24]	Cross-sectional	Hospital	P	Multinational	wP and aP	2011–2014	3451 (52)
Gill (2016) [34]	Cohort	Population	P	Zambia	wP	2015	775 (10)
Hallbauer^#^ (2016) [35]	Cross-sectional	Hospital	P	South Africa	aP	2008–2015	1259 (183)
Muloiwa^#^ (2016) [36]	Cross-sectional	Hospital	C, P	South Africa	aP	2011–2012	460 (41)
Nunes (2016) [37]	Clinical trial	Population	P	South Africa	aP	2011–2012	1644 (79)
Soofie (2016) [38]	Cross-sectional	Hospital	P	South Africa	aP	2015	1839 (42)
Zar (2016) [39]	Cohort	Population	P	South Africa	aP	2012–2014	284 (16)
du Plessis^#^ (2018) [40]	Surveillance	Hospital	C, P	South Africa	aP	2013–2015	990 (76)
**Eastern Mediterranean**							
Al-Bargish^#^ (1999) [41]	Cross-sectional	Hospital	C	Iran	wP	1996	133 (67)
Kakar (2009) [42]	Surveillance	Hospital	C	Afghanistan	wP	2006–2007	203 (7)
Ghanaie^#^ (2010) [43]	Cross-sectional	Population	C, P	Iran	wP	2007–2008	328 (27)
Bokhari^#^ (2011) [44]	Cross-sectional	Hospital	C, P	Pakistan	wP	2005–2009	802 (64)
Hajia (2012) [45]	Cross-sectional	Hospital	P	Iran	wP	2008–2011	138 (12)
Mughal (2012) [46]	Cross-sectional	Hospital	C	Pakistan	wP	2004–2006	700 (22)
Zouari^#^ (2012) [47]	Cross-sectional	Hospital	C, P	Tunisia	wP	2007–2011	599 (120)
Bahari (2013) [48]	Cross-sectional	Hospital	C	Iran	wP	2008–2012	156 (7)
Nikbin (2013) [49]	Cross-sectional	Hospital	C, P	Iran	wP	2009–2010	779 (100)
Saffar (2014) [50]	Cross-sectional	Hospital	P	Iran	wP	2008–2012	518 (43)
Sedaghat^#^ (2014) [51]	Cross-sectional	Hospital	C, P	Iran	wP	2004–2008	347 (30)
Benamrouche (2016) [52]	Surveillance	Hospital	C, P	Algeria	wP	2012–2013	246 (123)
Ghorbani (2016) [53]	Surveillance	Hospital	P	Iran	wP	2011–2013	3629 (239)
Omer (2016) [54]	Surveillance	Hospital	P	Pakistan	wP	2015–2016	2021 (8)
Katfy^#^ (2017) [55]	Cross-sectional	Hospital	C, P	Morocco	wP	2013–2015	156 (88)
Ben Fraji^#^ (2018) [56]	Cross-sectional	Hospital	C, P	Tunisia	wP	2007–2017	1844 (306)
Dumaidi (2018) [57]	Cross-sectional	Hospital	P	West Bank	wP	2004–2008	267 (130)
Mohammadzadeh (2018) [58]	Cross-sectional	Hospital	C, P	Iran	wP	2015–2016	184 (43)
**Europe**							
Lukić-Grlić^#^ (1999) [59]	Cross-sectional	Hospital	C, S	Croatia	wP	1988–1994	201 (2)
Daǧla (2004) [60]	Cross-sectional	Hospital	C	Turkey	wP	2001–2003	66 (2)
Aksakal (2007) [61]	Cross-sectional	Population	S	Turkey	wP	2004	307 (51)
Yıldırım (2008) [62]	Cross-sectional	Hospital	P, S	Turkey	wP	2005–2006	148 (16)
Medkova (2010) [63]	Cross-sectional	Unclear	C, P	Russian Fed.	wP	Unknown	172 (81)
Gürsel (2012) [64]	Cross-sectional	Hospital	C, P, S	Turkey	aP	2009–2010	51 (6)
Karlı (2013) [65]	Cross-sectional	Hospital	C, P	Turkey	aP	2008–2012	40 (6)
Uslu (2013) [66]	Cross-sectional	Hospital	P	Turkey	aP	2012	173 (48)
Dinu (2014) [67]	Cross-sectional	Hospital	C, P, S	Romania	aP	2012–2013	51 (14)
Karagül (2014) [68]	Cross-sectional	Hospital	C, P	Turkey	aP	2010–2011	214 (26)
Öksüz^#^ (2014) [69]	Cross-sectional	Hospital	C, P	Turkey	aP	2010–2013	410 (106)
Aslan (2016) [70]	Cross-sectional	Hospital	P	Turkey	aP	2013–2014	101 (20)
Goktas (2016) [71]	Cross-sectional	Hospital	P	Turkey	aP	2014–2015	845 (15)
Gökçe (2018) [72]	Cross-sectional	Hospital	P	Turkey	aP	2013–2016	172 (44)
**South-East Asia**							
Singh (1987) [73]	Cross-sectional	Hospital	C	India	wP	c.1986	560 (20)
Dahiya (2009) [74]	Cross-sectional	Hospital	C, P	India	wP	2007–2007	21 (2)
Barger-Kamate (2016) [24]	Cross-sectional	Hospital	P	Multinational	wP	2011–2014	749 (1)
Das (2016) [75]	Cross-sectional	Hospital	P	India	wP	2013–2014	180 (7)
Siriyakorn (2016) [76]	Cross-sectional	Hospital	P	Thailand	wP	2010–2011	76 (14)
Hughes^#^ (2017) [77]	Cohort	Population	P	Nepal	wP	2011–2014	2026 (17)
Chinthate (2018) [78]	Cross-sectional	Hospital	P	Thailand	wP	2016–2017	70 (7)
**The Americas**							
Cooper (1983) [79]	Surveillance	Hospital	C, S	St Lucia	wP	1981	10 (2)
Baptista (2006) [80]	Cross-sectional	Hospital	C	Brazil	wP	2003	287 (51)
Kowalzik (2007) [25]	Cross-sectional	Hospital	P	Multinational	wP and aP	2001–2004	181 (19)
Sandoval (2008) [81]	Cross-sectional	Population	P	Mexico	wP	2002–2003	61 (20)
Nieto Guevara (2010) [82]	Surveillance	Hospital	C, P	Panama	wP and aP	2001–2008	759 (178)
Astudillo (2011) [83]	Cross-sectional	Hospital	C, P	Colombia	wP	2006–2007	133 (45)
Leite (2012) [84]	Surveillance	Hospital	C	Brazil	wP	2006–2008	652 (132)
Ferronato (2013) [85]	Cohort	Hospital	C, P	Brazil	wP	2009–2012	57 (25)
Ochoa-Perez (2014) [86]	Surveillance	Hospital	C, P	Mexico	aP	2011–2012	147 (59)
Vaz-de-Lima (2014) [87]	Surveillance	Hospital	P	Brazil	wP	2009–2012	503 (66)
Castillo (2015) [88]	Cross-sectional	Hospital	P	Peru	wP	2010–2012	392 (155)
Pavic-Espinoza (2015) [89]	Cross-sectional	Hospital	P	Peru	wP	2009–2010	596 (114)
Pimentel (2015) [90]	Cross-sectional	Hospital	C, P	Brazil	wP	2010–2011	192 (10)
del Valle-Mendoza (2015) [91]	Cross-sectional	Hospital	P	Peru	wP	2010–2013	133 (51)
Bailon^#^ (2016) [92]	Cross-sectional	Hospital	C, P	Peru	wP	2012	840 (191)
Aquino-Andrade^#^ (2017) [93]	Cross-sectional	Hospital	P	Mexico	aP	2011–2014	286 (192)
Phadke (2018)^#^ [94]	Cross-sectional	Hospital	P	Guatemala	wP	2009–2012	301 (11)
del Valle-Mendoza (2018) [95]	Cross-sectional	Hospital	P	Peru	wP	2016–2017	88 (18)
**Western Pacific**							
Ong (1978) [96]	Cross-sectional	Hospital	C	Malaysia	wP	1974	65 (1)
Lin (2010) [97]	Cross-sectional	Hospital	C, P	China	wP	2008–2009	1001 (99)
Mi (2013) [98]	Cross-sectional	Hospital	P	China	wP	2011–2012	176 (51)
Ting (2013) [99]	Cross-sectional	Hospital	C, P	Malaysia	wP	2011	707 (275)
Huang (2014) [100]	Surveillance	Population	P	China	wP and aP	2010–2012	1022 (113)
Liu (2014) [101]	Cross-sectional	Hospital	C, P	China	wP	2013	148 (101)
Wang (2014) [102]	Cross-sectional	Hospital	C, P	China	wP and aP	2012–2013	313 (122)
Hu (2015) [103]	Cross-sectional	Hospital	P	China	aP	2013–2014	2536 (247)
Moriuchi (2017) [104]	Cross-sectional	Unclear	P	Cambodia	wP	2008–2016	651 (82)
Sadiasa (2017) [105]	Cross-sectional	Hospital	P	Philippines	wP	2012–2015	1152 (34)

*C* culture, *P* polymerase chain reaction, *S* paired serology, *wP* whole cell vaccine, *aP* acellular vaccine

^#^Includes *Bordetella parapertussis.* NB. Cooper, Strebel and Al-Bargish conducted in outbreak settings

Study designs included three clinical trials, four cohorts, 62 cross-sectional and 13 surveillance studies. There were 71 studies published in English, four in Mandarin, three each in Spanish and Turkish and one in Persian. Three studies by Cooper and Fitch, Strebel et al. and Al-Bargish et al. were conducted in outbreak settings [28, 41, 79].

In total, the studies originated from 37 countries, representing all six WHO regions (Additional file 2). Nineteen (51%) of the countries represented were upper middle-income, while 11 (30%) and seven (19%) were lower middle- and low-income countries, respectively. Five countries contributed 28 (49%) of the studies (Turkey = 11, Iran = 9, South Africa = 8, China = 6 and Brazil = 6). Sixty-four (78%) studies had epidemiological data for *Bordetella pertussis* only while 18 (22%) studies investigated for both *Bordetella pertussis* and *Bordetella parapertussis*. The most frequently used vaccine over the period the included studies were conducted was wP in 72/88 (82%) settings either on its own (*n* = 66) or in combination with aP (*n* = 6). In 16 (18%) settings, aP was the only vaccine in use.

Data from a study by Zouari et al. was considered only for the purpose of estimating mortality but not for estimation of disease burden as its data overlapped with that of Ben Fraj et al. who reported cases over a longer period but did not report on mortality [47, 56].

### Prevalence of pertussis

**Fig. 2:**
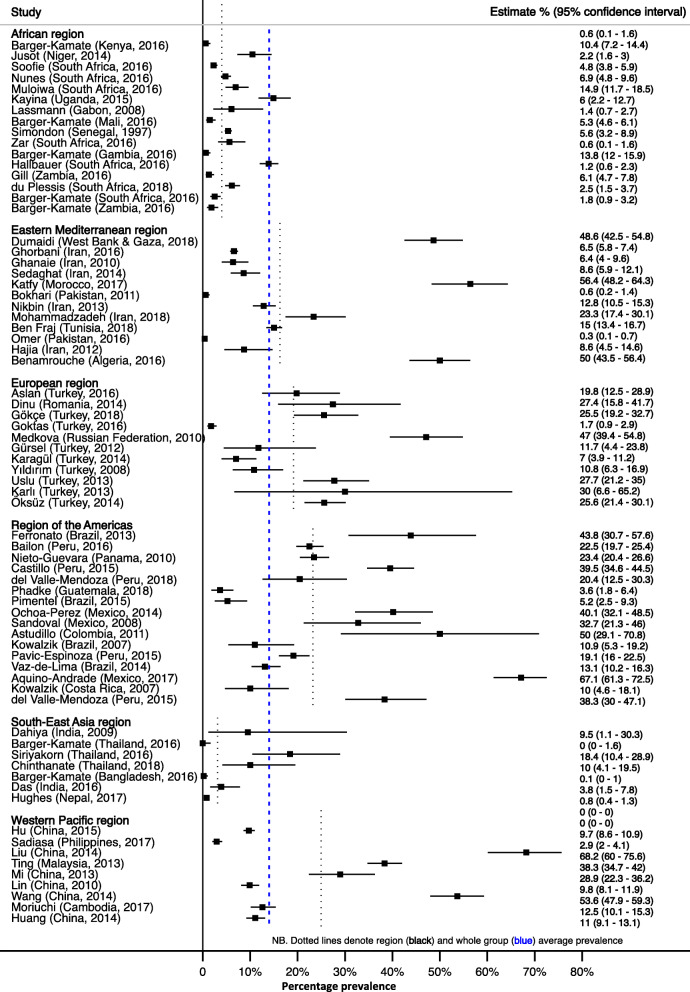
Prevalence of polymerase chain reaction-confirmed *Bordetella pertussis*. Dotted lines show subgroup and whole group average estimates

The median prevalence of PCR-confirmed disease due to *Bordetella pertussis* was 11% (IQR, 5–27%; *n* = 43,696, 64 studies) (Fig. 2 and Additional file 3). PCR prevalence differed across WHO regions ranging from a median of 4% (IQR 4–10%) in South-East Asia to a median of 22% (IQR 12–40%) in the Region of the Americas, *P* = 0.001. In one multinational study, conducted in countries in the Africa and South-East Asia regions, Barger-Kamate et al. found an increased risk for pertussis in African countries with an adjusted odds ratio of 8.8 (*P* = 0.03) [24].

**Fig. 3:**
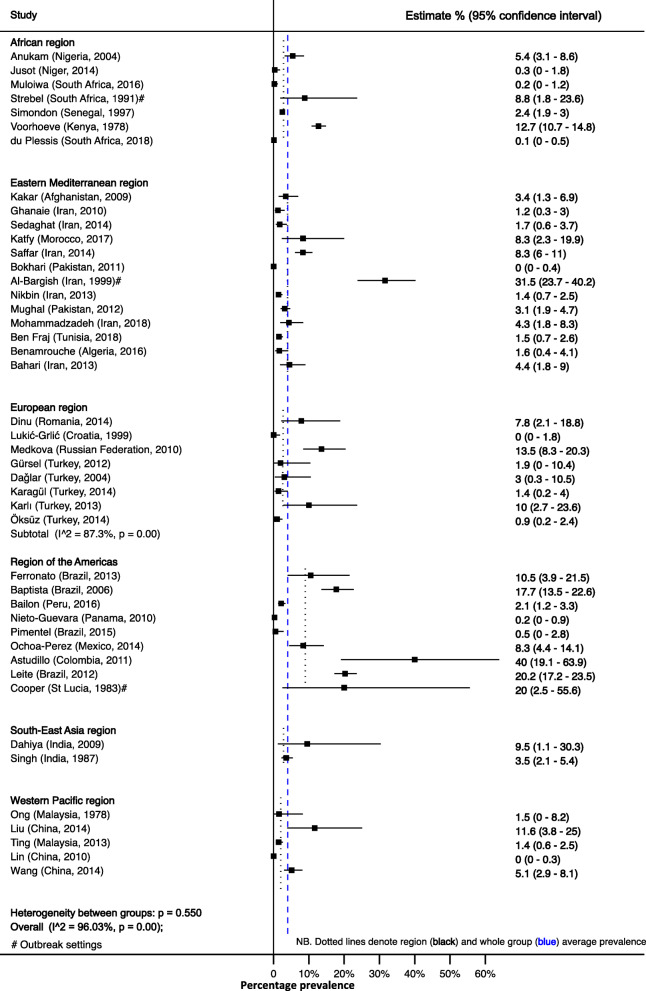
Prevalence of culture-confirmed *Bordetella pertussis*. Dotted lines show subgroup and whole group average estimates

The median prevalence of culture-confirmed Bordetella *pertussis* was 3% (IQR 1–9%) (*n* = 18,868, 44 studies). The point prevalence was similar across WHO regions, *P* = 0.1380 (Fig. 3 and Additional file 2).

Confirmation of *Bordetella pertussis* using paired serology showed a median of 17% (IQR 3–23%; *n* = 4912, 9 studies). Only three WHO regions were represented, and the median prevalence was 3% (IQR 3–13%) in the African region and 17% (95% CI, 13–26%) in the European region, while the one country, St Lucia, representing the Region of the Americas had a prevalence of 44% (95% CI, 14–79%), *P* = 0.1309 (Additional file 3 and Additional file 4).

The prevalence of confirmed *Bordetella parapertussis* infection using any of the three confirmatory methods was 1% (IQR, 0–2%; *n* = 12,062, 18 studies). The Eastern Mediterranean region was noted to have the highest prevalence with a median 2% (IQR 0–7%) with one study from the same region having a prevalence of 19% (95% CI 13–26%) [41] (Additional file 5).

The prevalence of *Bordetella pertussis* differed in the same population depending on the method of laboratory confirmation used. On average, culture underestimated prevalence by 85% (RR = 0.15, 95% CI, 0.10–0.22) compared to PCR in the 29 (*n* = 14,315) studies that used both methods (Additional file 6).

**Fig. 4:**
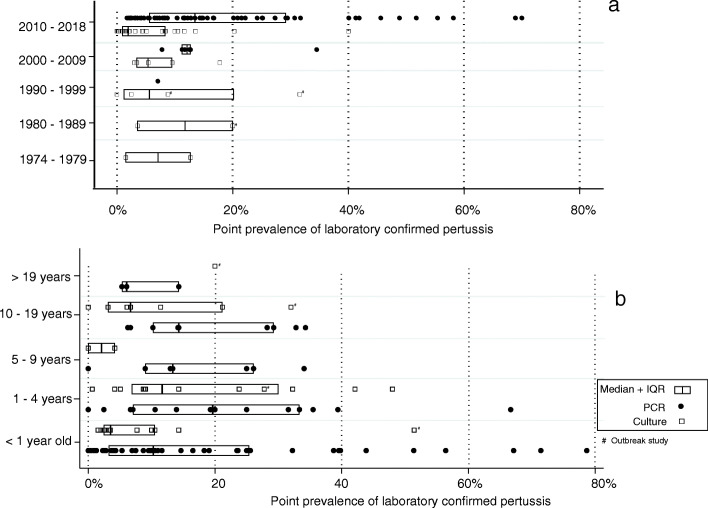
Distribution of point prevalence of polymerase chain reaction- and culture-confirmed pertussis by period (**a**) and age group (**b**)

Pertussis prevalence declined in the 1990s from the levels seen in the 1970s and 1980s. A slight increase was noted since the period after 2000 (Fig. 4a). Huang et al. reported a 26-fold increase in confirmed adult pertussis between 2010 and 2014 in China [100]. There was sufficient information in 48 studies to estimate age prevalence by age group. The lowest prevalence was noted in individuals older than 19 years with a median prevalence of 6% (IQR 5–14%). After the high prevalence noted below 5 years of age, the risk declined in late childhood (6 to 10 years) but increased again in adolescents who showed the highest prevalence of all groups with a median prevalence of 20% (IQR 14–32%) (Fig. 4b).

Pertussis prevalence was also stratified by the study setting (hospital versus population based) as well as by the type of vaccine used in settings where the included studies were conducted. The prevalence of pertussis in hospital-based studies had a median of 10% (IQR 4–25%) compared to population-based studies which reported a median prevalence of 6% (IQR 3–13%). There was an overlap in the distribution of prevalence of confirmed pertussis in populations using aP only [median 10% (IQR 6–28%)] compared to populations using some wP [median 10% (IQR 3–20%)]. The overlap remained even when prevalence was stratified by the diagnostic method. In the only included clinical trial comparing the two vaccine types, Simondon et al. found aP vaccine efficacy against all confirmed pertussis to be 85% (95% CI, 66–93%) and that of wP to be 96% (95% CI, 86–99%) [29].

### Incidence of pertussis

Population-level incidence rates of pertussis and hospitalisation were reported by some of the authors, but these could not be independently verified as the requisite population data were not available (Table 2). In addition, a majority of the authors reported point estimate incidences with no confidence intervals. Where data was available for different age groups (Voorhoeve et al., Nieto Guevara et al., Saffar et al. and Ochoa-Perez et al.), the incidence was always highest in infancy [26, 50, 82, 86]. The highest incidence of 15,900/100000 was reported in Kenyan infants between 1974 and 1977 [26]. In addition to *Bordetella pertussis* incidence, Ghanaie et al. reported a separate incidence of 2 per 100,000 for *Bordetella parapertussis* [43]. Contacts were reported to have an incidence of 0.69/100000 in Benamrouche et al.’s study [52].

**Table 2:** Population and hospitalisation incidence rates of *Bordetella pertussis*

Study (year)	Incidence	Age ranges	Country
Voorhoeve (1978) [26]	3800/100000	All ages	Kenya
Strebel^#^ (1991) [28]	187/100000	6 months to 5 years	South Africa
Simondon (1997) [29]	119/100000	2 months to 15 years	Senegal
Sandoval (2008) [81]	500/100000	12 to 15 years	Mexico
Ghanaie (2010) [43]	318/100000	6 to 14 years	Iran
Nieto Guevara^#^ (2010) [82]	144/100000	All ages	Panama
Uslu (2013) [66]	0.9/100000	< 5 years	Turkey
Huang (2014) [100]	23.52/100000	All ages	China
Jusot (2014) [32]	820/100000	< 5 years	Niger
Ochoa-Perez (2014) [86]	2.3/100000	0 to > 18 years	Mexico
Saffar (2014) [50]	4.92/100000	0 month to 25 years	Iran
Benamrouche (2016) [52]	1.04/100000	All ages	Algeria
Gill (2016) [34]	520/100000	< 1 year	Zambia
Muloiwa^#^ (2016) [36]	526/100000	< 13 years	South Africa
Omer (2016) [54]	247/100000	< 1 year	Pakistan
Soofie (2016) [38]	220/100000	< 1 year	South Africa
Giayetto (2017) [106]	4.53/100000	All ages	Argentina
Ben Fraj (2018) [56]	134/100000	< 5 years	Tunisia

^#^Incidence represents hospitalisation rates for pertussis

Time-denominated rates were recorded for three studies. Gill et al. reported a rate of 2.4 (95% CI, 1.2–4.2) cases per 1000 infant-months with the highest rate of 3.5 cases/1000 infant-months noted between birth and 6 weeks, while Hughes et al. reported a rate of 13.3 (95% CI, 7.7–21.3) cases per 1000 infant-years [34, 77]. Nunes et al.’s data gave an overall rate of 4.9 per 1000 person-months, which differed between infants (5.7/1000) and mothers (4.3/1000) as well as by HIV status as reported below [37].

### Risk of pertussis in HIV exposed and infected

Ten studies, all from the Africa region, investigated the impact of HIV status on the risk of pertussis. The incidence rate of pertussis was 7.4/1000 infant-months in HEU infants and 5.5/1000 in HUU infants in the study by Nunes et al., while the rates in HIV+ and HIV uninfected mothers were 6.8 and 3.9/1000, respectively [37]. Gill et al. reported RR 1.8 (95% CI, 95% CI 0.5–6.9) in HEU infants compared to HUU. The incidence of *Bordetella pertussis*-associated hospitalisation was 2.9 (95% CI, 1.8–4.5) and 1.9 (95% CI, 1.3–2.6) per 1000 in HIV-exposed and HIV-unexposed infants, respectively, in a study by Soofie et al. The reported 4.8% case fatality rate in Soofie et al. was only due to deaths in HIV-exposed infants [38]. In the study by Hallbauer et al., there was insufficient data to estimate stratum-specific rates, but HIV+ cases, who made 14% of the study sample, accounted for 22 (19%) of the 113 pertussis cases with known HIV status [35]. A gradual increase in risk of pertussis was reported in a study by Muloiwa et al. in which the risk of pertussis was 5.4% in HUU, 10.9% in HEU and 15.8% in HIV+ [36].

**Fig. 5:**
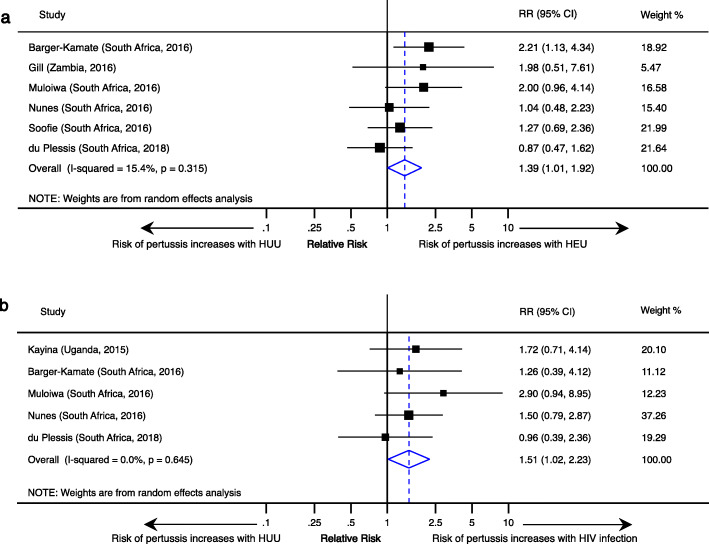
Meta-analysis of the relative risk of pertussis comparing HIV-unexposed uninfected (HUU) to HIV-exposed uninfected (HEU) (**a**) and HIV infected (**b**)

There was sufficient data to do a meta-analysis comparing risk of pertussis in HUU with HEU and HIV+ in six and five studies, respectively. Compared to HUU, HIV+ and HEU individuals had a RR 1.51 (95% CI, 1.02–2.23) and RR 1.4 (95% CI, 1.01–1.92) for confirmed pertussis, respectively (Fig. 5). The highest risk of pertussis was reported by Anukam et al., in a cohort of wP-vaccinated HIV-infected adolescents who were not on antiretroviral therapy with RR 22.8 (95% CI, 6.9–75.1) [30]. This study was not included in the HIV meta-analysis as it was an obvious outlier composed of individuals not on treatment which seemed to show excessive risk for pertussis. All other studies involved HIV+ individuals on antiretroviral therapy.

### Mortality rate and case fatality rate of pertussis

**Fig. 6:**
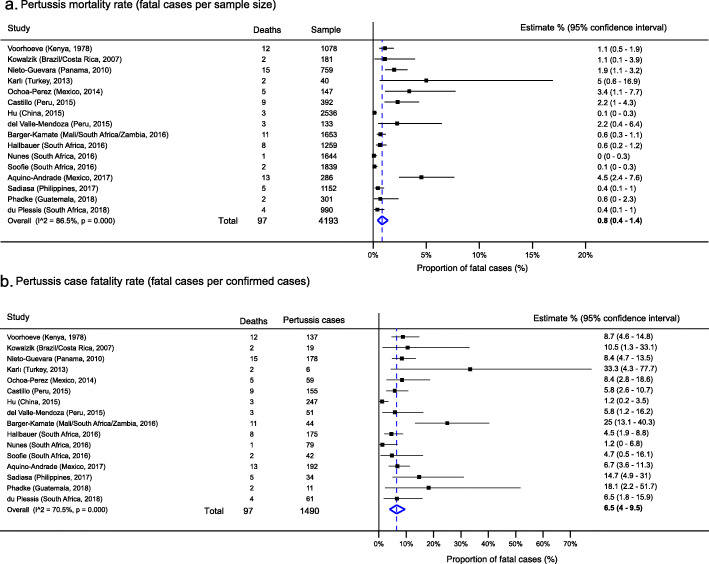
Mortality and case fatality rate of confirmed pertussis

A total of 97 pertussis-related deaths out of 1490 confirmed cases were reported in 16 studies (*n* = 14,390) representing 13 countries. All deaths were associated with *Bordetella pertussis* with none attributed to *Bordetella parapertussis.* From the 16 studies, the overall mortality rate was 0.8% (95% CI, 0.4–1.5%) with a pertussis case fatality rate of 5.5% (95% CI, 3.3–6.1%) (Fig. 6). When only infants were considered (13 studies), the case fatality rate was 7.2% (95% CI, 3.6–11.8%) in the studies reporting deaths.

All children who died were younger than 5 years and the majority were younger than 6 months of age. Almost all deaths occurred under 1 year of age with only one study (Voorhoeve et al.) reporting pertussis deaths after the second year of life (*n* = 5) [26].

### Quality of the included studies

Using the modified tool published with the protocol for this systematic review, we found all the included studies to be of high quality [15]. This is because components of the quality index score, such as laboratory confirmation and availability of raw denominator and numerator data formed part of the inclusion criteria, which automatically excluded poor-quality studies. Similarly, studies had moderate to low risk of attrition and selection bias.

## Discussion

This study comprehensively reports on the burden of confirmed pertussis over a 45-year period (1974–2018) in LMICs. This period starts in 1974 with the inception of EPI. The prevalence of confirmed pertussis disease differed in the same study depending on the method of laboratory confirmation with PCR showing the greatest diagnostic sensitivity as expected [5]. Most cases were due to *Bordetella pertussis*. *Bordetella parapertussis* was less common and did not have any reported fatalities associated with it. The study indicates that pertussis deaths are significantly high in LMICs with a disproportionate case fatality rate in young infants. Secondly, the meta-analysis shows that HIV has a significant impact in the burden of pertussis in settings where the burden of HIV is high.

Not surprisingly, the findings from our study agree with those from some HICs: the highest incidence of pertussis was in infants and the greatest pertussis-specific mortality in children younger than 6 months [107–109]. Moreover, we also noted the increase in the prevalence of pertussis in adolescents similarly described in highly vaccinated cohorts from HICs [110–112]. The noted decline in adulthood may indicate protection following the natural boosting in adolescence. Worryingly, the pooled case fatality rate of nearly 6% exceeds the less than 4% estimated by WHO for developing countries [2]. As noted, this is even higher when only infants are considered.

The prevalence data presented in this systematic review suggests that LMICs may also be experiencing a resurgence of pertussis as noted in HICs. Both the GPI and WHO advocate for the strengthening of surveillance systems as a key component in the control of pertussis [110, 113]. Currently, surveillance of pertussis in LMICs is suboptimal. As a result, there are many gaps in accurate pertussis epidemiological data which we observed in this study. The review indicates that the choice of laboratory case confirmation influences the quantification of pertussis disease burden within the same setting. This is an important finding that suggests that the use of PCR to confirm pertussis should be prioritised in LMICs. The higher sensitivity of PCR is more likely to capture the true burden of pertussis and give a better understanding of the global epidemiological pattern of the disease across different settings than any other method. In contrast, culture, recognised as the diagnostic gold standard, missed on average 85% of cases identified by PCR in the studies that used both methods in this systematic review. Higher culture prevalence estimates than in other studies in a similar WHO group were noted when the studies were conducted before a vaccination programme was fully established (Voorhoeve et al.), during an outbreak (Cooper and Fitch [79]; Strebel et al.; and Al-Bargish) and when the study included secondary attack data of close contacts (Astudillo et al.) [26, 28, 41, 83].

A number of authors reported incidences although population denominators could not be independently verified as these went largely unreported. The rates were quite high compared to HICs and showed no pattern of decline over the period in which the incidences were reported.

An unexpected finding was the significant overlap in the prevalence of pertussis noted with different vaccines in use. Most LMICs use wP vaccines in contrast with the predominant use of aP vaccines in HICs. In general, wP vaccines are regarded as offering better protection against pertussis [114]. Despite the predominant use of wP in the reviewed studies, we noted a steady increase in confirmed pertussis in studies reporting after 2000. This suggests that the observed resurgence of pertussis noted in HIC may only be partly explained by the change of vaccine from wP to aP in these countries. Another possible explanation for the increase may be the increase in the use of PCR for case confirmation—all the included studies conducted after 2010 used PCR as the primary method of confirming cases. The increase in the observed prevalence coincides with the use of these molecular techniques.

Although data are limited by the number of countries reporting HIV status, there is strong evidence from the meta-analysis showing that the risk of pertussis is increased in HIV+ and HEU individuals. The risk of pertussis was increased by 50% and 40% respectively in the two groups compared to HUU. With the exception of the study by du Plessis et al., all studies showed an increased risk of pertussis incidence or prevalence associated with HIV infection or exposure [40]. In addition, there was a higher risk of hospitalisation and deaths related to pertussis in HIV-exposed or HIV-infected infants [34, 37, 38]. In considering their pertussis control strategies, LMICs, which have the biggest burden of HIV, need to take into account this increased risk associated with HIV exposure and infection [8].

Our study is largely limited by paucity of data, especially longitudinal data for each included country as well as vaccine coverage and number of vaccine doses received in the specific population studied. The available data was disproportionately provided by only a few upper middle-income countries. A further limitation is the case detection that may have been affected by the variability of PCR assays used. In particular, this is noted with respect to our inability to assess the sensitivity and specificity of each assay. Regardless, our results will encourage generation of more epidemiological studies on pertussis in LMICs, while in the meantime, assisting policy makers in disease control planning.

## Conclusions

Immunisation remains suboptimal with vaccine coverage with the three infant doses (DPT3) remaining low in LMIC in general and in low-income countries, in particular [115]. Even where vaccine coverage with DPT3 is acceptable, the doses are not often received in a timely manner, undermining the protective effect of the vaccine on young infants [116, 117]. Unlike in HIC countries in which re-emergence of pertussis may be related to low and delayed vaccine coverage secondary to vaccine hesitancy, in LMIC, this usually reflects the lack of administrative capacity [118]. This study indicates an urgent need to review and strengthen the existing pertussis control programmes in LMICs to target children, adolescents and HIV-exposed and HIV-infected groups. In addition, the study highlights the need to urgently consider measures to reduce the high infant mortality rate, with specific consideration for maternal vaccination. There is now strong evidence to showing that immunisation of pregnant women, even when given as early as the second trimester, is safe for both the mother and the foetus and induces sufficient transplacental antibodies to protect the young infant still too young to complete primary immunisation schedule for pertussis [119, 120]. In their recommendation, the GPI in addition to prioritising surveillance and increasing overall vaccine coverage made this an urgent area of action for LMICs [6]. Encouragingly, we noted a substantial increase in the number of studies published in the last 8 years of the period under review (2010–2018), possibly reflecting recent increase in interest and funding for pertussis research in LMICs.

## Supplementary information

**Additional file 1: Table**: Strategy used to search for literature in MEDLINE (Via Pubmed).

**Additional file 2.** Country and year of included studies with confirmed pertussis shown by World Health Organisation region.

**Additional file 3.** Distribution of point prevalence of confirmed pertussis by World Health Organisation region and confirmation method [PCR = polymerase chain reaction].

**Additional file 4 **Prevalence of paired serology confirmed *Bordetella pertussis.* Dotted lines show subgroup and whole group average estimates.

**Additional file 5 **Prevalence of polymerase chain reaction and culture confirmed *Bordetella parapertussis.* Dotted line shows group average estimate [# Culture confirmed].

**Additional file 6.** Meta-analysis of relative detection rates of polymerase chain reaction (PCR) and culture in confirming Bordetella pertussis infection.

## Data Availability

All data generated or analysed during this study are included in this published article and in the reference list provided, all of which are in the public domain.

## Abbreviations

aP: Acellular vaccine
CDC: Centers for Disease Control and Prevention
CIs: Confidence intervals
EPI: Expanded Programme on Immunisation
GPI: Global Pertussis Initiative
HEU: HIV-exposed uninfected
HICs: High-income countries
HIV+: HIV infected
HUU: HIV-unexposed uninfected
IQR: Interquartile range
LMICs: Low- and middle-income countries
PCR: Polymerase chain reaction
RR: Relative risks
WHO: World Health Organization
wP: Whole cell vaccine
