# Supplementary material for: The burden of laboratory-confirmed pertussis in low- and middle-income countries since the inception of the Expanded Programme on Immunisation (EPI) in 1974: a systematic review and meta-analysis
Source: BMC Med. 2020 Aug 28;18:233. doi: 10.1186/s12916-020-01699-3 (PMC7453720; doi:10.1186/s12916-020-01699-3)
Supplement: Supplementary file 1 — Additional file 1: Table: Strategy used to search for literature in MEDLINE (Via Pubmed). [file 12916_2020_1699_MOESM1_ESM.docx]

| Table: Strategy used to search for literature in MEDLINE (Via Pubmed) | |
| --- | --- |
| Query No. | Search term |
| #1 | Pertussis (MeSH) OR whooping cough (MeSH) |
| #2 | Bordetella pertussis OR B. pertussis OR Bordetella parapertussis OR B. parapertussis |
| #3 | #1 OR #2 |
| #4 | Burden OR epidemiology OR incidence OR prevalence OR case* |
| #5 | #3 AND #4 |
| #6 | (Afghanistan OR Albania OR Algeria OR American Samoa OR Angola OR Armenia OR Armenian Azerbaijan OR Bangladesh OR Belize OR Benin OR Byelarus OR Byelorussian OR Belarus OR Belorussian OR Belorussia OR Bhutan OR Bolivia OR Bosnia OR Herzegovina OR Hercegovina OR Botswana OR Republic of Botswana OR Brazil OR Brasil OR Bulgaria OR Burkina Faso OR Burkina Fasso OR Upper Volta OR Burundi OR Urundi OR Cambodia OR Khmer Republic OR Kampuchea OR Cameroon OR Cameroons OR Cameron OR Camerons OR Cape Verde OR Cabo Verde OR Central African Republic OR Chad OR China OR Colombia OR Comoros OR Comoro Islands OR Comores OR Mayotte OR Congo OR Republic of Congo OR Zaire OR Costa Rica OR Cote d'Ivoire OR Ivory Coast OR Cuba OR Djibouti OR French Somaliland OR Democratic Republic of Congo OR DRC OR Zaire OR Dominica OR Dominican Republic OR East Timor OR East Timur OR Timor Leste OR Ecuador OR Egypt OR United Arab Republic OR El Salvador OR Eritrea OR Equatorial Guinea OR Ethiopia OR Fiji OR Gabon OR Gabonese Republic OR Gambia OR Georgia Republic OR Georgian Republic OR Georgia) |
| #7 | (Ghana OR Gold Coast OR Greece OR Grenada OR Guatemala OR Guinea OR Guinea-Bissau OR Guiana OR Guyana OR Haiti OR Honduras OR India OR Maldives OR Indonesia OR Iran OR Iraq OR Jamaica OR Jordan OR Kazakhstan OR Kazakh OR Kenya OR Kiribati OR Korea OR Kosovo OR Kyrgyzstan OR Kirghizia OR Kyrgyz Republic OR Kirghiz OR Kirgizstan OR Lao PDR OR Laos OR Lebanon OR Lesotho OR Basutoland OR Liberia OR Libya OR Macedonia OR Madagascar OR Malagasy Republic OR Malaysia OR Malaya OR Malay OR Sabah OR Sarawak OR Malawi OR Nyasaland OR Mali OR Marshall Islands OR Mauritania OR Mauritius OR Agalega Islands OR Mexico OR Micronesia OR Moldova OR Moldovia OR Moldovian OR Mongolia OR Montenegro OR Morocco OR Ifni OR Mozambique OR Myanmar OR Myanma OR Burma OR Namibia OR South-West Africa OR Nauru OR Nepal OR Nicaragua OR Niger OR Nigeria OR Pakistan OR Palestine OR Papua New Guinea OR Paraguay OR Peru OR Philippines OR Philipines OR Phillipines OR Phillippines OR Romania OR Rumania OR Roumania) |
| #8 | (Rwanda OR Ruanda OR Saint Kitts OR St Kitts OR Nevis OR Saint Lucia OR St Lucia OR Saint Vincent OR St Vincent OR Grenadines OR Samoa OR Samoan Islands OR Navigator Island OR Navigator Islands OR Sao Tome OR Sao Tome and Principe OR São Tomé OR Senegal OR Serbia OR Montenegro OR Sierra Leone OR Sri Lanka OR Ceylon OR Solomon Islands OR Somalia OR Somaliland OR Sudan OR South Sudan OR South Africa OR Republic of South Africa OR Suriname OR Surinam OR Swaziland OR Syria OR Syrian Arab Republic OR Tajikistan OR Tadzhikistan OR Tadjikistan OR Tadzhik OR Tanzania OR United Republic of Tanzania OR Thailand OR Tuvalu OR Togo OR Togolese Republic OR Tonga OR Tunisia OR Turkey OR Turkmenistan OR Turkmen OR Uganda OR Ukraine OR USSR OR Soviet Union OR Union of Soviet Socialist Republics OR Russian Federation OR Russia OR Uzbekistan OR Uzbek OR Vanuatu OR New Hebrides OR Venezuela OR Vietnam OR Viet Nam OR West Bank OR West Bank and Gaza OR Gaza OR Yemen OR Yugoslavia OR Zambia OR Zimbabwe OR Rhodesia) |
| #9 | #6 OR #7 OR #8 |
| #10 | #5 AND #9 |
