# Supplementary figures and images for: The burden of laboratory-confirmed pertussis in low- and middle-income countries since the inception of the Expanded Programme on Immunisation (EPI) in 1974: a systematic review and meta-analysis
Source: BMC Med. 2020 Aug 28;18:233. doi: 10.1186/s12916-020-01699-3 (PMC7453720; doi:10.1186/s12916-020-01699-3)

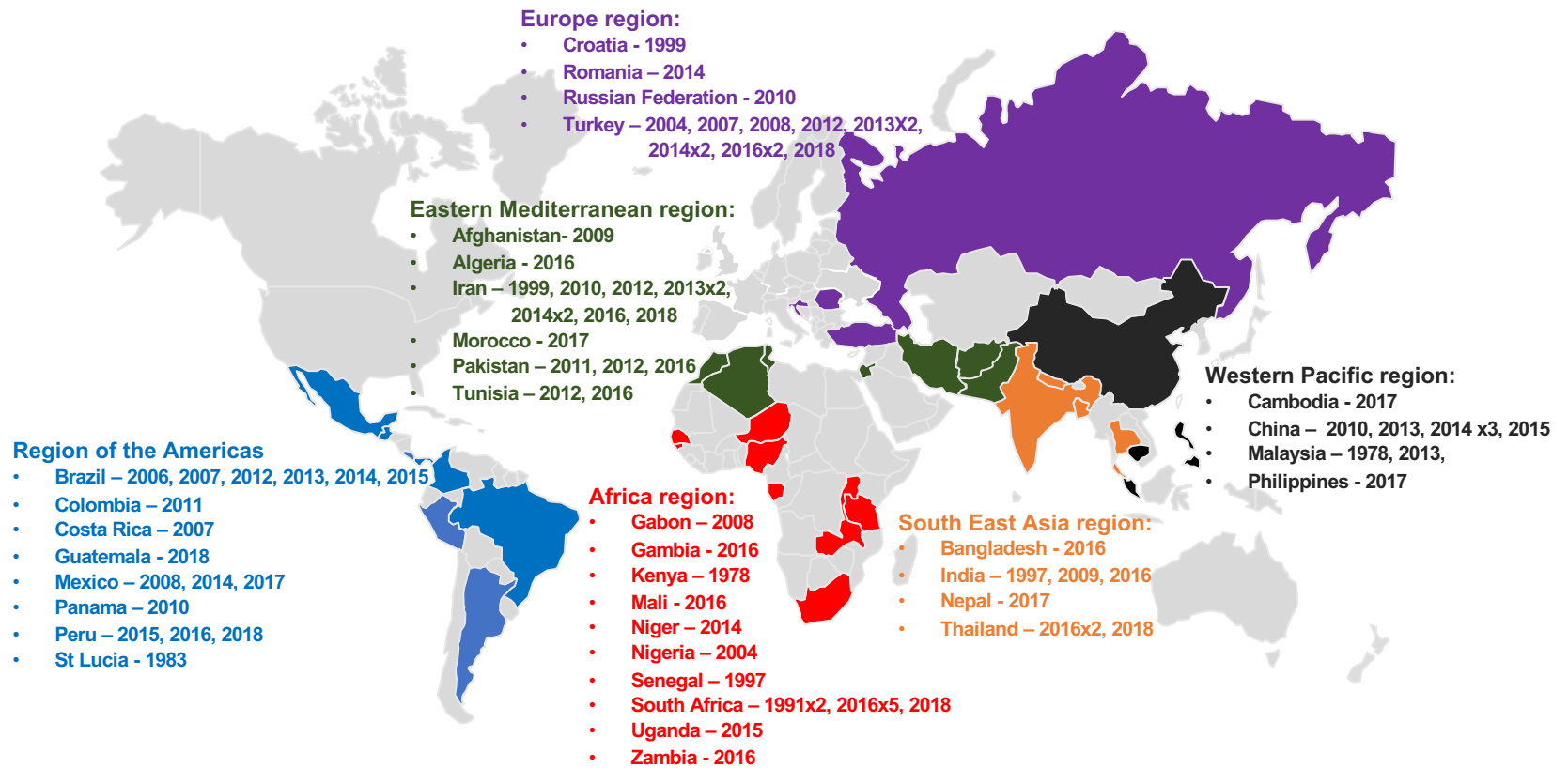

Supplement: Supplementary file 2 — Additional file 2. Country and year of included studies with confirmed pertussis shown by World Health Organisation region. [file 12916_2020_1699_MOESM2_ESM.pdf]

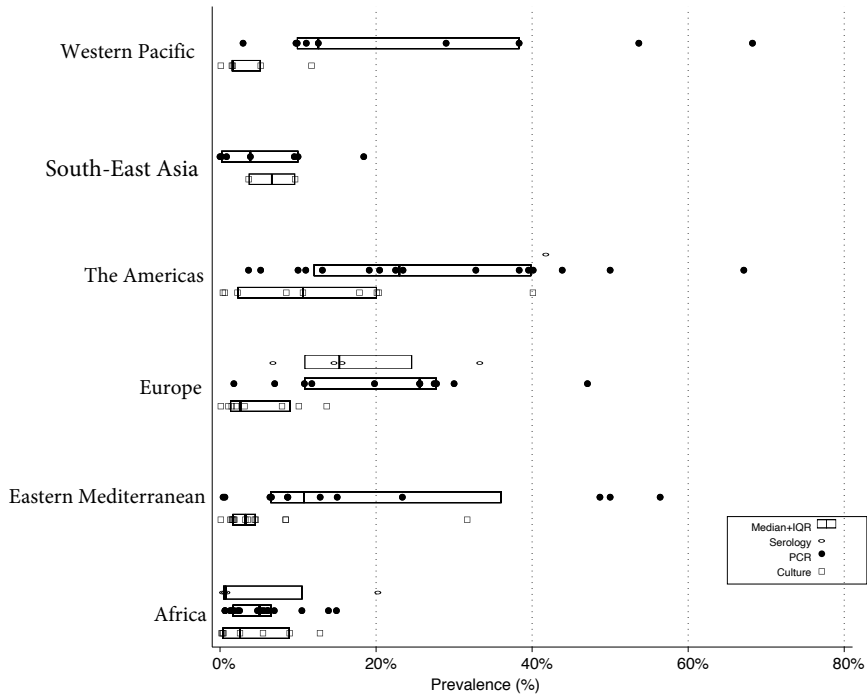

Supplement: Supplementary file 3 — Additional file 3. Distribution of point prevalence of confirmed pertussis by World Health Organisation region and confirmation method [PCR = polymerase chain reaction]. [file 12916_2020_1699_MOESM3_ESM.pdf]

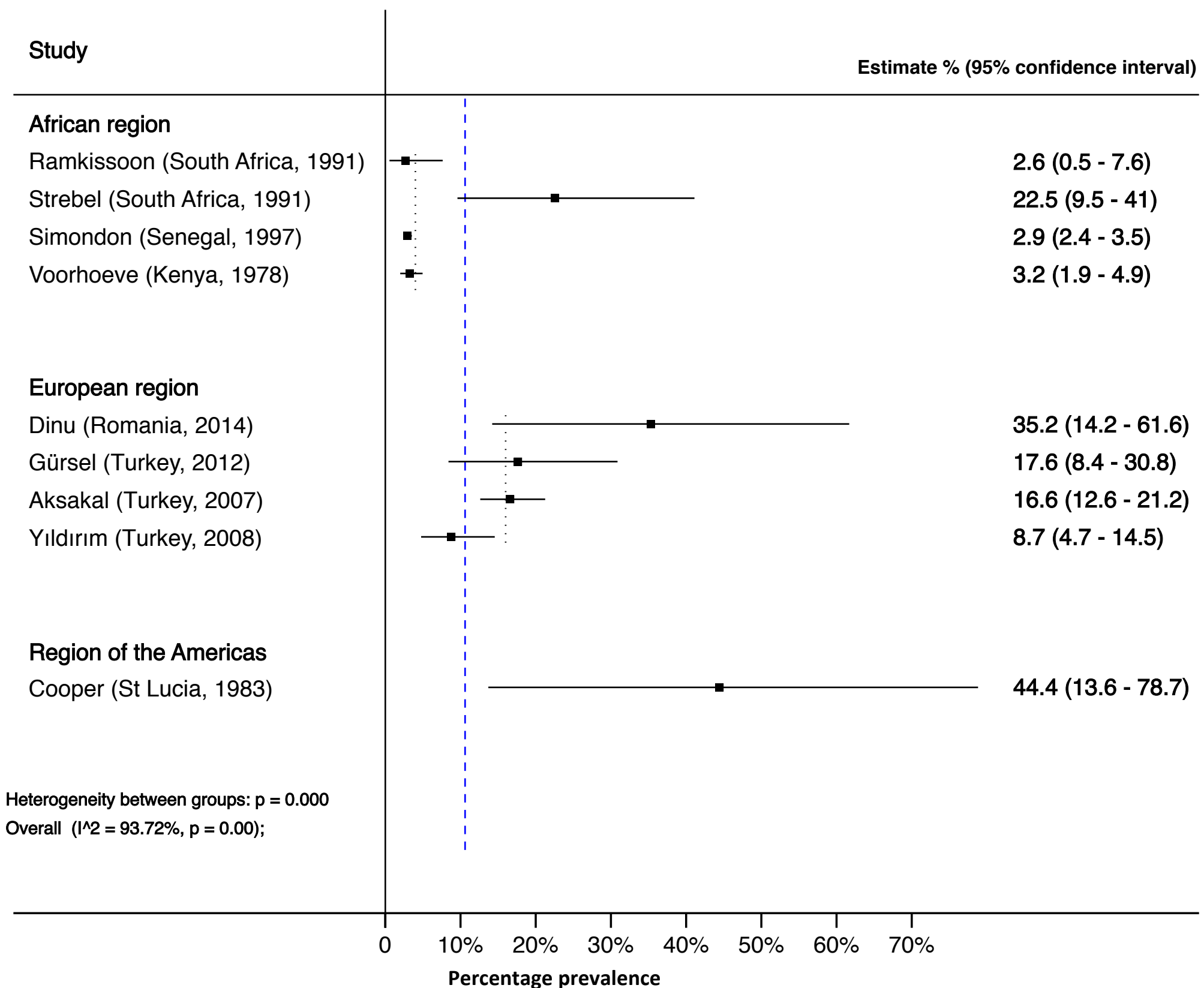

Supplement: Supplementary file 4 — Additional file 4 Prevalence of paired serology confirmed Bordetella pertussis. Dotted lines show subgroup and whole group average estimates. [file 12916_2020_1699_MOESM4_ESM.pdf]

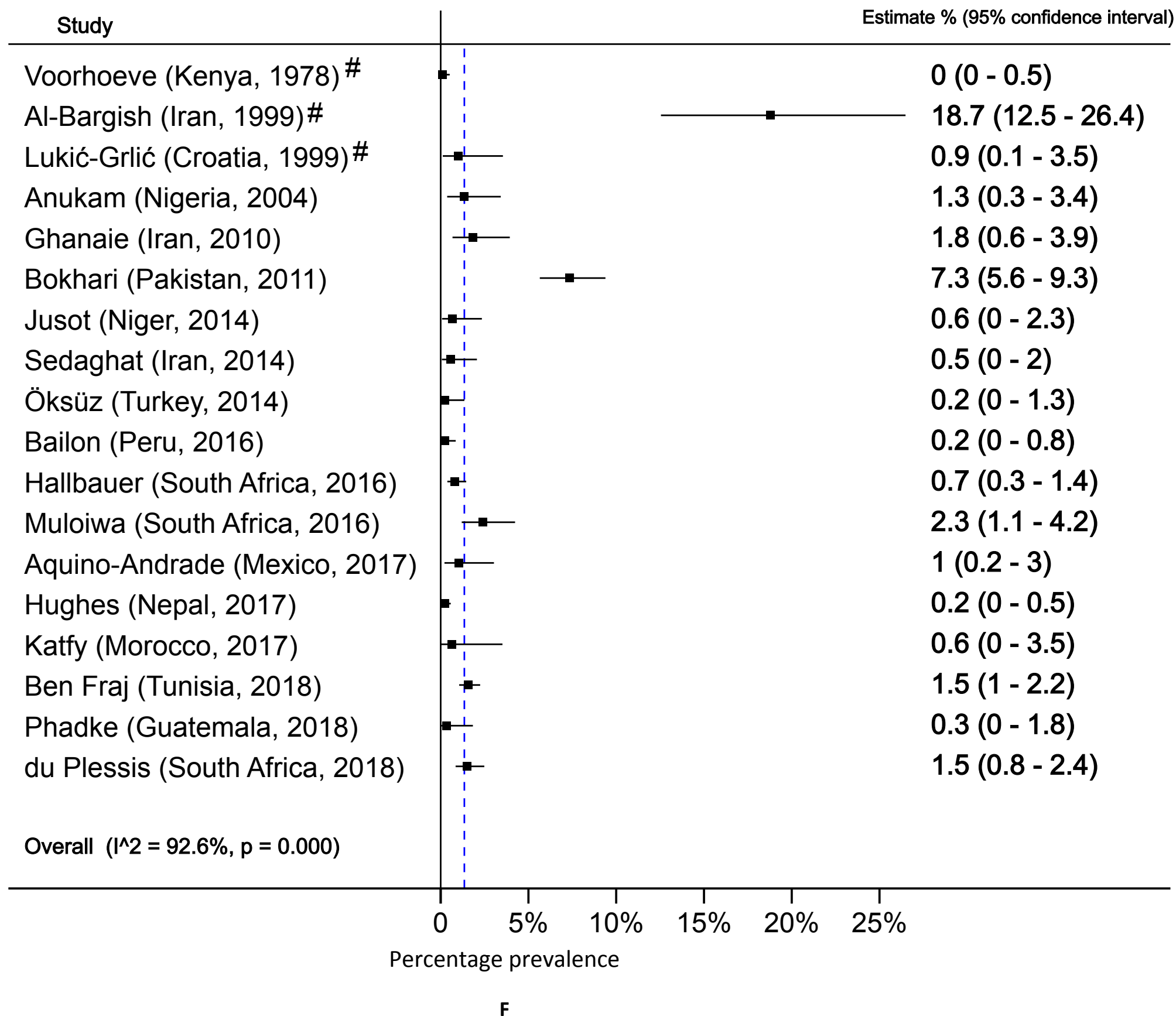

Supplement: Supplementary file 5 — Additional file 5 Prevalence of polymerase chain reaction and culture confirmed Bordetella parapertussis. Dotted line shows group average estimate [# Culture confirmed]. [file 12916_2020_1699_MOESM5_ESM.pdf]

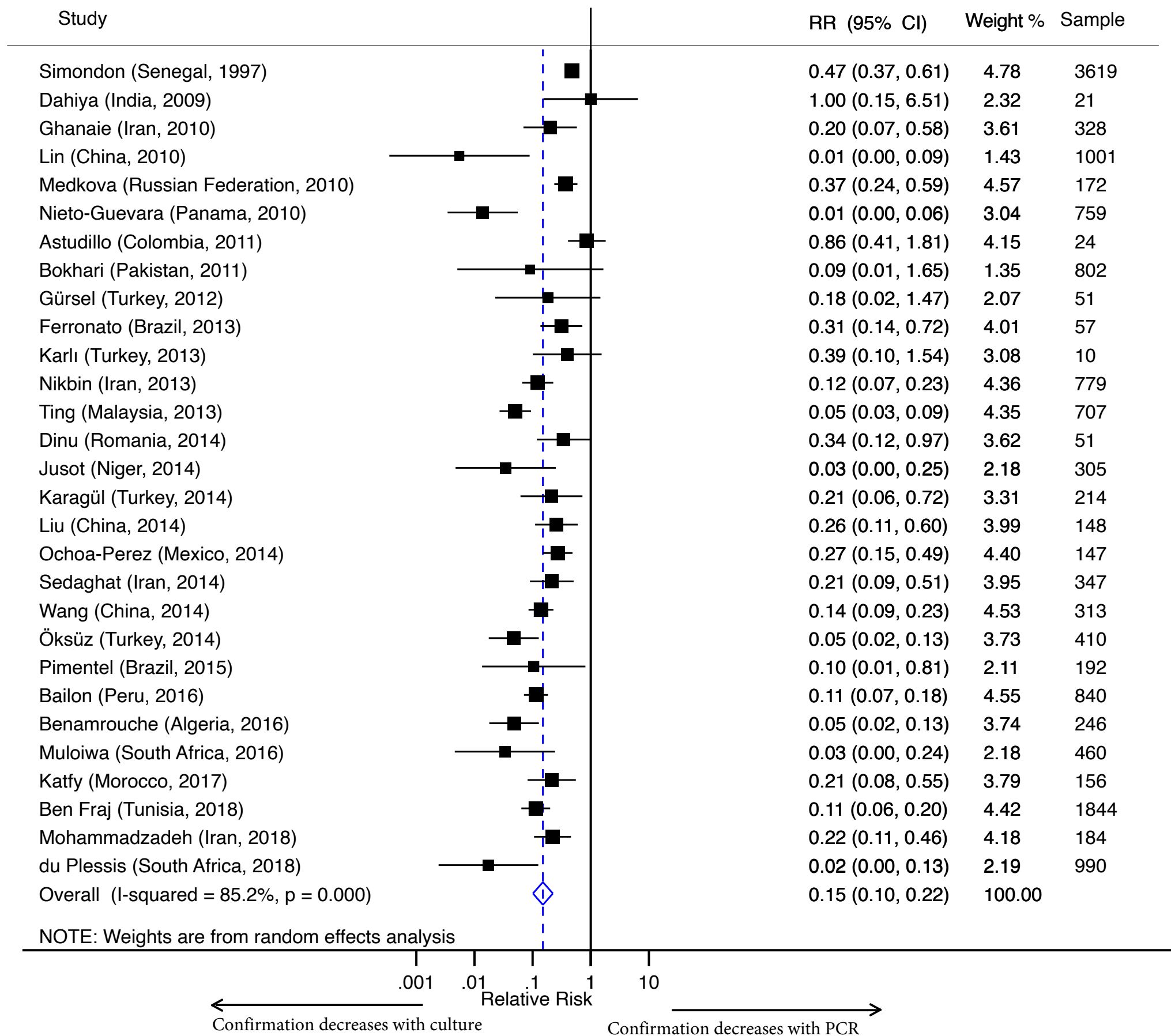

Supplement: Supplementary file 6 — Additional file 6. Meta-analysis of relative detection rates of polymerase chain reaction (PCR) and culture in confirming Bordetella pertussis infection. [file 12916_2020_1699_MOESM6_ESM.pdf]
